# Dual branch attention network for image super-resolution

**DOI:** 10.1038/s41598-025-97190-1

**Published:** 2025-08-08

**Authors:** Yiwei Hu, Yisu Ge, Mingming Qi, Shuhua Xu

**Affiliations:** 1 College of Computer Science and Artificial Intelligence, Wenzhou University, Wenzhou, 325000 China; 2 School of Data Science and Artificial Intelligence, Wenzhou University of Technology, Wenzhou, 325000 China

**Keywords:** Super-resolution, CNN, Transformer, Self-attention, Dictionary, Energy science and technology, Mathematics and computing

## Abstract

The advancement of deep convolutional neural networks (CNNs) has resulted in remarkable achievements in image super-resolution methods utilizing CNNs. However, these methods have been limited by a narrow perceptual field and often require a high number of parameters and computational complexity, making them unsuitable for resource-constrained devices. Recently, the Transformer architecture has shown significant potential in image super-resolution due to its ability to perceive global features. Yet, the quadratic computational complexity of self-attention mechanisms in these Transformer-based methods leads to substantial computational and parameter overhead, limiting their practical application. To address these challenges, we introduce the Dual Branch Attention Network (DBAN), a novel Transformer model that integrates prior knowledge from traditional dictionary learning with the global feature perception capabilities of Transformers, enabling image super-resolution. Our model features a ”token dictionary” mechanism that uses auxiliary labeling to provide external prior information, enhancing cross-attention and self-attention computations while maintaining a linear relationship between computational complexity and image size. We also propose a Feature Aggregation Module (FAM) that efficiently extracts local contextual information and performs channel feature fusion, substantially enhancing the model’s performance and efficiency. By reasonably arranging the number of modules and the depth of the network, we reduce the complexity of the model. Extensive experiments have demonstrated that our DBAN achieves excellent performance.

## Introduction

Image super-resolution reconstruction technology, commonly referred to as ISR, is utilized to enhance the details of low-resolution (LR) images and produce high-resolution (HR) outputs. It significantly elevates the visual quality and intricacy of images. This technology not only elevates the clarity of images but also assists us in overcoming the technical constraints of outdated equipment, thereby enabling them to generate higher-quality image outputs. The application of ISR technology is pivotal in the realm of medical imaging^1^. It aids doctors in diagnosing diseases with greater precision by elevating image resolution, particularly in X-rays, MRI^2^, and CT scans, where superior resolution signifies the ability to discern more minute lesions and structures. Furthermore, ISR technology holds significant importance in remote sensing, as it amplifies the resolution of satellite imagery, facilitating enhanced monitoring of land use changes, environmental shifts, and crop growth. Video surveillance stands as another significant application area for ISR technology. By elevating the resolution of images captured by surveillance cameras, it enables more efficient safety monitoring, traffic supervision, and public safety management. The digital entertainment industry, particularly in sectors such as animation, gaming, and film production, benefits significantly from ISR technology. This technology enhances visual effects and significantly improves the user experience. Researchers have proposed various methods to achieve ISR, mainly categorized into three types: interpolation-based methods^3^, reconstruction-based methods^4^, and learning-based methods^5^. Interpolation-based methods estimate high-frequency details of images using mathematical algorithms, such as nearest neighbor interpolation, bilinear interpolation, and bicubic interpolation. Reconstruction-based strategies involve establishing a degradation model for the image and utilizing prior knowledge to restore its details. Learning-based techniques, especially the latest progress in deep learning, train models to grasp the mapping relationship between LR and HR images, thus accomplishing super-resolution reconstruction.

**Fig. 1:**
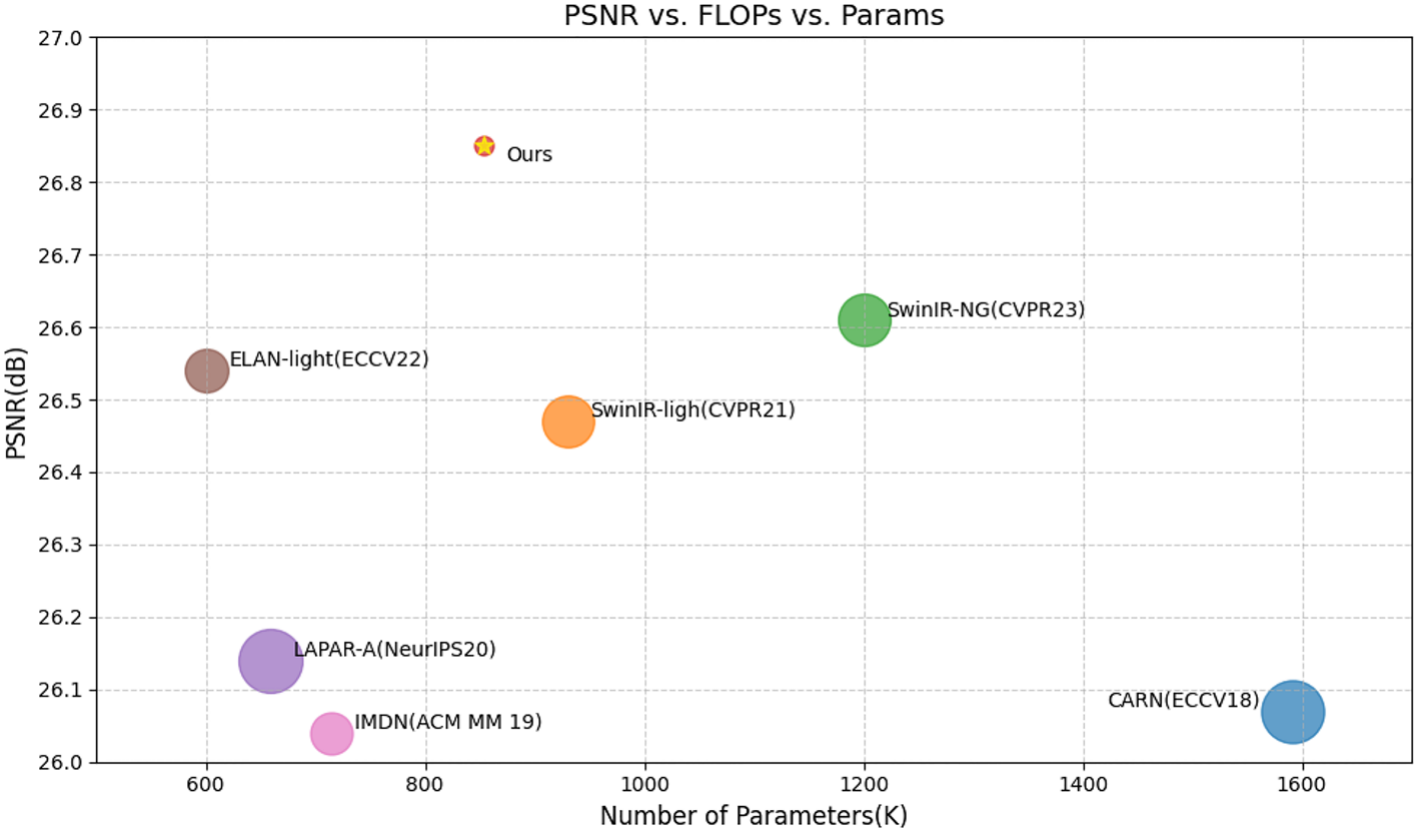
A comparison of model complexity and performance between our proposed DBAN model and other techniques on the Urban100 dataset for $\times$4 super-resolution. The circle size indicates the computational complexity in terms of floating-point operations. Our approach offers a more favorable balance between model intricacy and reconstruction performance.

Over the past few years, the swift advancement of hardware technology has empowered the training of deep learning-based super-resolution (SR) methods, like CNNs^6–8^, on extensive datasets. However, these convolutional networks focus solely on modeling local feature dependencies, neglecting the critical establishment of global dependencies, which significantly hinders model performance. As an illustrative case, the Enhanced Deep Residual Networks (EDSR)^9^, despite its optimization of the conventional CNN architecture—specifically, by eliminating the batch normalization layer and employing residual learning—still possesses a large number of parameters totaling 43 million. This not only amplifies the model’s complexity but also leads to a notably prolonged processing time for a single image, approximately 0.5 s under identical hardware conditions. Such latency is impractical for real-time applications. Additionally, the performance of Transformers^10–13^ has consistently outperformed that of convolutional neural networks in various low-level visual tasks. The essence of the Transformer’s superiority is attributed to its self-attention (SA) mechanism^14–20^, which facilitates the creation of global dependencies. This capability counters the inherent drawbacks in CNN-based approaches, highlighting the importance of global feature interaction for high-quality image reconstruction. Despite the recent progress in image super-resolution, challenges persist. Transformer-based^21–24^ approaches feature self-attention mechanisms that entail a quadratic level of computational complexity, including SwinIR^25^, necessitates a trade-off between achieving optimal SR accuracy and controlling computational expenses. To manage computational load, previous methods have limited attention computation to local windows. For instance, SwinIR^25^ employs a $7\times 7$ local window, reducing computational complexity but concurrently restricting the receptive field, which in turn affects the SR performance. The Omni Aggregation Network (OAN)^26^ was introduced to enhance SR performance through multi-scale feature fusion within a fully aggregated network framework. However, OAN^26^ still faces constraints due to the local window attention mechanism, resulting in suboptimal handling of long-distance dependencies. Although OAN^26^ improves performance through multi-level feature aggregation, it does not entirely surmount the limitations that local windows impose on receptive fields, which in turn affects the overall effectiveness of SR. Studies have revealed that expanding the window size can widen the receptive field and enhance SR outcomes, but this also amplifies the challenges associated with high dimensionality. This situation emphasizes the requirement for a strategy that can model long-range dependencies efficiently, transcending the limitations of local windows. Furthermore, traditional SR methodologies for images typically rely on generic algorithms that do not adapt to the specific characteristics of individual images. Segmenting images based on their content categories, rather than rectangular local window partitioning strategies, may be more advantageous for the SR process and could lead to higher-quality image reconstruction.

Fueled by the insights gained from previous challenges, we present DBAN as a solution for image super-resolution. In the conceptualization of DBAN, we have embraced the fundamental principles of classical dictionary learning^27^ and applied them to the field of super-resolution, introducing an innovative mechanism termed the “token dictionary.” This mechanism is designed to enhance cross-attention and self-attention computations within image processing and is distinguished by three significant features. Firstly, the token dictionary acquires a suite of auxiliary tokens that encapsulate common image structures, and by employing a cross-attention mechanism, it seamlessly integrates external prior knowledge into the image processing pipeline. This strategy ensures efficient processing, preserving a linear correlation between computational complexity and the scale of the image. Secondly, the token dictionary leverages global information to refine its content by selectively activating specific tokens. It also establishes long-range connections by distilling specific image information through a reverse attention mechanism, which is crucial for capturing long-distance dependencies within the image. Lastly, the token dictionary transcends the limitations of local window segmentation by enabling the use of all analogous image segments to enhance labeling. This is achieved through category self-attention computations that capitalize on the similarities between images and dictionary labels, thereby more effectively preserving and amplifying the structural information inherent in images. Beyond the Transformer block, the feed-forward network (FFN^28^) operates to capture features utilizing a dense layer. Nonetheless, this network fails to consider spatial details in its feature extraction process. To address this shortcoming, we have developed a spatial gating feedforward network (SGFN). This innovative network incorporates a spatial gate (SG) module within the sequence of two dense layers that constitute the FFN^28^. The SG module functions as a straightforward regulatory element, leveraging deep convolution alongside element-wise multiplication to refine the input features. The SG module operates by segmenting the input features into two distinct parts along the channel axis, which allows for individual convolution and multiplication processes. By doing so, the SG module enhances the FFN with further nonlinear characteristics that are spatially aware, thereby mitigating the issue of redundant channels. This integration of spatial awareness into the FFN^28^ through the SGFN leads to a more nuanced and effective feature extraction process. With the aid of SGFN, the Triple Attention Block (TAB) facilitates feature aggregation within the block. Concurrently, drawing inspiration from SAFMN^29^, we have designed a convolutional module for feature extraction that can dynamically select the most representative features. In summary, we found that DBAN achieves a superior balance between model complexity and super-resolution performance, as depicted in Fig. 1. Our contributions can be summarized as follows:

- We have designed a novel dual-branch multi-task and efficient image SR model, DBAN. Our DBAN aggregates features within blocks and between branches to achieve strong representation capabilities.
- We introduce the concept of dictionary learning, which provides prior information for each image label through a set of auxiliary labels, and effectively summarizes the feature information of the entire image through a set of parallel cross-attention modules and a spatial gating feed-forward mechanism. Moreover, we introduce a FAM module designed to concurrently extract local contextual information and carry out channel feature fusion.
- We carried out both quantitative and qualitative assessments of the proposed method using benchmark datasets. The outcomes showed that our DBAN not only effectively balances accuracy with model complexity, but also attains state-of-the-art performance in terms of both reconstruction accuracy and visual quality.

## Related work

### Image super-resolution based on deep learning

Deep convolution-based methods have historically played a pivotal role in ISR. More recent advancements have focused on feature extraction and fusion techniques to enhance the accuracy and efficiency of single image super-resolution (SISR) models. As an illustration, PF-OA^30^ offers a state-of-the-art technique employing both 1D and 2D convolutional kernels to derive orientation-aware characteristics. It employs a channel attention mechanism to adaptively choose the most informative features. This progressive feature fusion scheme effectively integrates hierarchical features, resulting in a compact yet potent CNN-based model designed for high-quality SISR. Meanwhile, DiVANet^31^ introduces a directional variance attention mechanism aimed at capturing long-range spatial dependencies and simultaneously exploiting inter-channel dependencies for more discriminative feature representations. The novel incorporation of attention mechanisms alongside residual blocks in DiVANet^31^ enhances the preservation of finer details, showcasing its superiority over state-of-the-art models in terms of both restoration accuracy and computational efficiency. HCANet^32^ achieves superior feature extraction through capturing long-range dependencies and neighborhood spectral correlations. PlainUSR^33^ achieved a 2.9-fold speedup without compromising performance by utilizing reparameterized MBconv blocks.

### Vision transformer

Inspired by the success of vision transformers (ViT)^34–36^ in diverse advanced visual challenges, researchers are now delving into the application of Transformer-based models for basic visual tasks. In the field of ISR, these methods have surpassed CNN-based models. For example, SwinIR^25^ retains the architecture of the Swin Transformer^37^, utilizing spatial window self-attention and shift operations. ELAN^38^ employs grouped multi-scale self-attention, while ART^39^ and OmniSR^40^ utilize sparse attention, and GRL^41^ uses anchored self-attention–all strategies aimed at expanding the receptive field and achieving better results. SSR^42^ segments the image into non-overlapping small blocks, employs a pyramid structure to identify blocks of interest across various scales, and exclusively reconstructs deep features for these designated blocks. This trend indicates that Transformer-based methods are increasingly supplanting CNN-based methods in the image domain, continually enhancing the performance of ISR. Nevertheless, owing to the intrinsic computational complexity of self-attention mechanisms, these Transformer-based methods incur significant computational and parameter overheads. Moreover, they require a large amount of training data to reach peak performance. This presents a significant constraint in practical application scenarios, especially when resources are scarce, thus posing a considerable limitation in such contexts.

## Method

In this section, we first describe the SR problem and subsequently present the architecture of our proposed method. After that, we delve into the specific details of the core components that constitute DBAN.

### Method description

Image super-resolution involves reconstructing corresponding HR images from LR images. In this context, we define LR images as $I_x$, typically obtained through the following degradation:

1 $$\begin{aligned} I_x = H(I_y; \delta ), \end{aligned}$$

where $I_y$ represents the HR image before degradation, and $H(\cdot )$ represents the degradation mapping function. $I_y$ maps to LR image $I_x$ through the degradation process, $\delta$ is a parameter of the degradation process,which generally encompasses factors such as scaling and noise. Usually, only degraded LR images $I_x$ are provided in SR tasks where the degradation mapping function is unknown. And it may also be affected by factors such as sensor noise, anisotropic degradation, compression artifacts, and speckle noise under different real-world conditions. To simplify this degradation mapping model, most SR algorithms simply represent it as a single downsampling process. It is defined as $\downarrow s$ (i.e., downsampling operation with a scaling factor of *s*), as follows:

2 $$\begin{aligned} H(I_y; \delta ) = (I_y \otimes k) \downarrow s, \{k,s\} \subset \delta , \end{aligned}$$

where $I_y \otimes k$ represents convolution with fuzzy kernel k. Usually, most datasets utilized in SR tasks employ the aforementioned simplified degradation model to construct LR images. These datasets utilize common bicubic interpolation downsampling operations and anti-aliasing techniques to generate LR and HR pairs, making them suitable for training sets and general benchmark datasets.

**Fig. 2:**
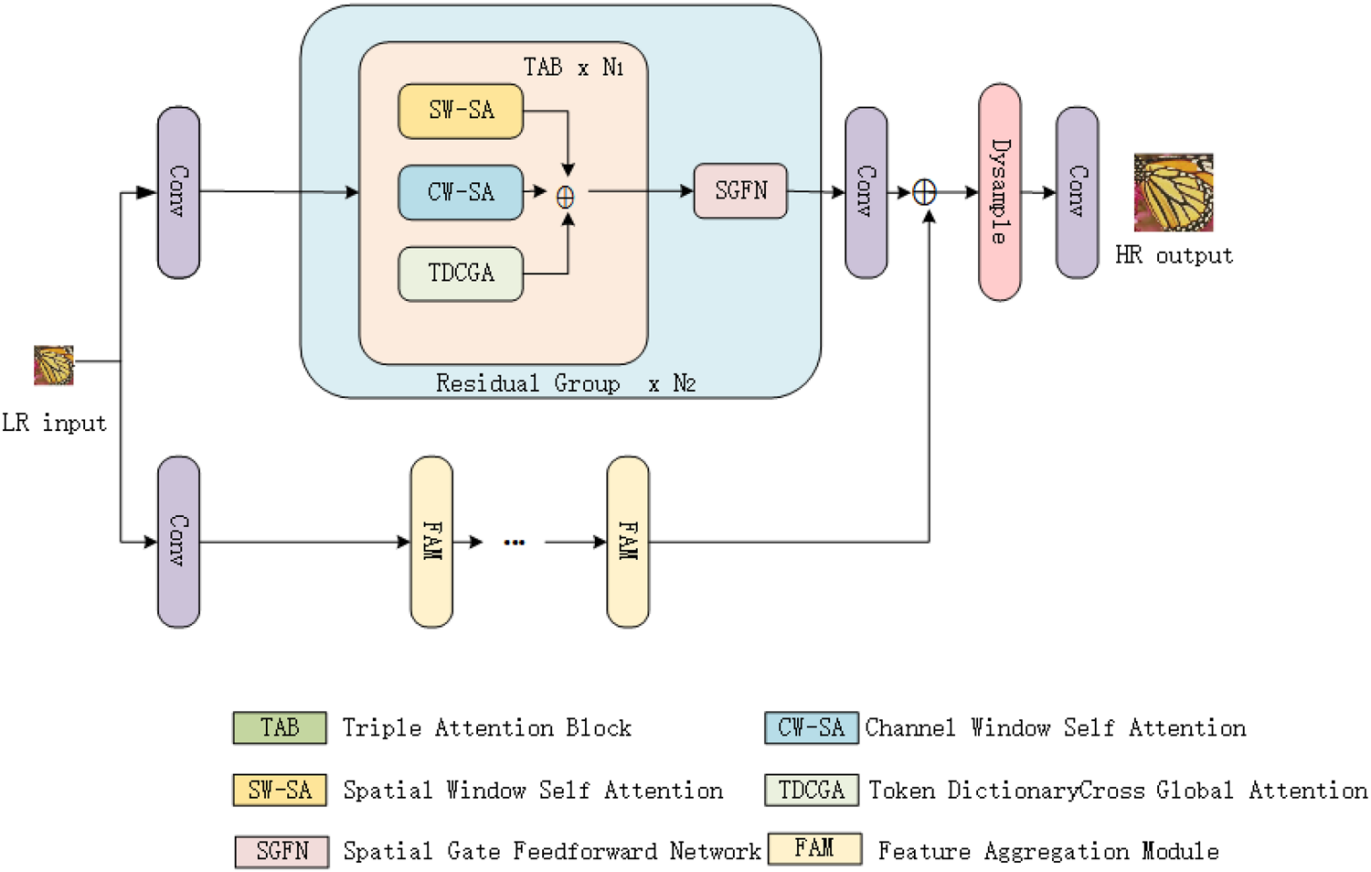
The architecture of the proposed DBAN model. Our model architecture comprises four parts: (1) shallow feature extraction; (2) deep feature extraction; (3) feature aggregation module; (4) reconstruction.

### Overall architecture

As depicted in Fig. 2, the proposed DBAN architecture mainly includes four sections: a module for extracting shallow features, another for deep feature extraction, a feature aggregation component, and a module for reconstructing HR images. We will define $I_x \in R^{h\times w\times 3}$ as the input LR image, where h and w are the height and width of the image, 3 represents the channel dimension of the image. Therefore, $H_{LF}(I_x)$ represents the shallow feature extraction module, which includes a $3\times 3$ convolutional layer to extract shallow features $F_{LF} \in R^{h\times w\times c}$ (c represents the feature dimension after the convolutional layer), $H_{LF}(\cdot )$ uses the convolutional layer to map the input LR image to a higher dimensional feature space.

3 $$\begin{aligned} F_{LF} = H_{LF}(I_x), \end{aligned}$$

Subsequently, the shallow features $F_{LF}$ are processed by the deep feature extraction module to obtain the deep features $F_{DF1} \in {R}^{h \times w \times c}$. This module is composed of multiple residual groups (RGs) stacked together, totaling $N_2$. Additionally, to ensure training stability, a residual strategy is employed within the module. Each RG comprises $N_1$ triple attention blocks (TABs). At the same time, shallow features are fed into FAM for feature fusion $F_{DF2} \in {R}^{h \times w \times c}$, and then the final deep features $F_{DF}$ are obtained by fusing the features of the two branches $F_{DF1}$ and $F_{DF2}$.

4 $$\begin{aligned} F_{DF} = F_{DF1} + F_{DF2}, \end{aligned}$$

Finally, we reconstruct the HR output image $I_{HR} \in {R}^{H_{out} \times W_{out} \times 3}$ through the reconstruction module, where $H_{out}$ is the height of the output image and $W_{out}$ represents the width of the output image. In this module, the DySample^43^ method is utilized for upsampling the deep features. Convolutional layers are utilized to gather features prior to and following upsampling.

5 $$\begin{aligned} I_{HR} = H_{RC}(F_{DF}), \end{aligned}$$

where $H_{RC}(\cdot )$ represents the reconstruction module.

**Fig. 3:**
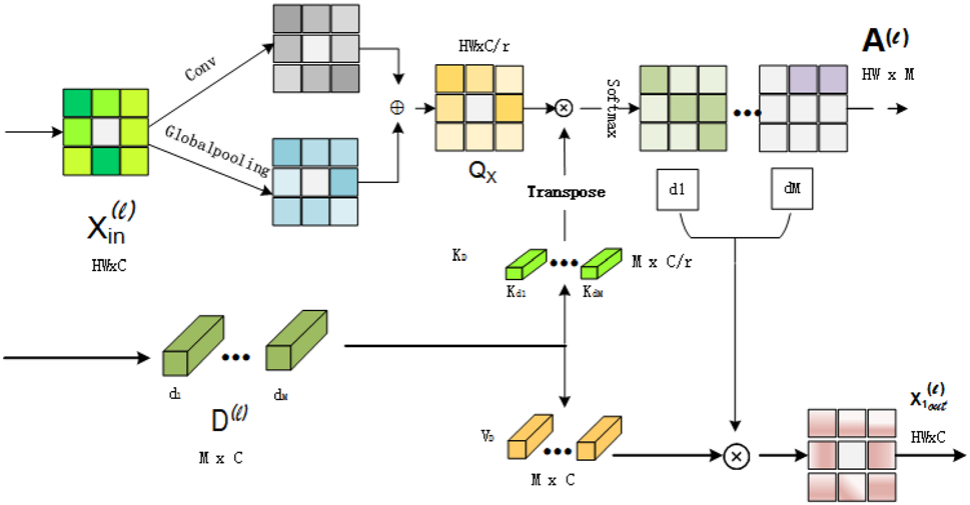
The proposed token dictionary cross global attention (TDCGA).

### Triple attention module

#### Token dictionary cross global attention

Within this section, we provide an in-depth look at our newly proposed token dictionary cross global attention block, as depicted in Fig. 3. In contrast to conventional multi-head self-attention (MSA) mechanisms that generate queries, keys, and values based on the input features themselves. Our goal is to initialize an additional dictionary as a grid parameter to introduce external query priors during the training phase. In traditional dictionary-based cross-attention methods, QueryToken mainly relies on the global information of input feature X. However, local features also contain a large amount of detailed information, that is particularly important for SR tasks. We extract local features through convolution operation before generating query tokens, and integrate them with global features to more effectively leverage both local and global information, thereby enhancing the model’s performance. Specifically, before generating the query token $Q_x$, we first extract local features through convolution operations. Next, we extract global features through global feature extraction operations. Then we use weighted summation operation to fuse local features and global features, generating comprehensive features $X_{combine}$.

6 $$\begin{aligned} X_{local}= Conv{_3\times _3}(X), \end{aligned}$$

7 $$\begin{aligned} X_{global}= GlobalPooling(X), \end{aligned}$$

8 $$\begin{aligned} X_{combine}= \alpha X_{local} + (1-\alpha ) X_{global}, \end{aligned}$$

where $Conv_{3\times 3}$ represents $3\times 3$ convolution, GlobalPooling represents the global average pooling operation, $\alpha$ is a learnable parameter used to balance the contributions of local and global features. Finally, we generate query tokens $Q_X$ using comprehensive features $X_{combine}$. In order to maintain consistency and integrity, $K_D$ and $V_D$ are still generated through dictionaries $D \in R^{M \times d}$, where M denotes the number of tokens in the dictionary, and d denotes the feature dimension of each token without involving the combination of local and global features.

9 $$\begin{aligned} Q_x = X_{combine} W_Q ,\quad K_D = D W_K, \quad V_D = D W_V , \end{aligned}$$

where $W_Q \in R^{\frac{d \times d}{r}}$, $W_K \in R^{\frac{d \times d}{r}}$, $W_V \in R^{d \times d}$ represent linear transformations for query tokens, key dictionary tokens, and value dictionary tokens, respectively. To maintain lower computational costs, we set $M<N$. The feature dimension of key dictionary tokens is reduced by a factor of 1/r to decrease model size and complexity, where r denotes the reduction ratio. Subsequently, we utilize the key dictionary and value dictionary to improve query tokens via cross attention computation.

10 $$\begin{aligned} & S = simcos(Q_x, K_D)/\tau , \end{aligned}$$

11 $$\begin{aligned} & A = Softmax(S), \end{aligned}$$

12 $$\begin{aligned} & TDCGA(Q_x, K_D, V_D) = AV_D, \end{aligned}$$

where $\tau$ is a learnable parameter used to adjust the range of similarity values. Simcos($Q_x$, $K_D$) represents the cosine similarity between two tokens calculated, the generated similarity graph $S \in R^{N \times M}$ describes the similarity between the query tokens and the key dictionary token. Where N represents the number of query tokens, and M represents the number of tokens in the dictionary. We use normalized cosine distance instead of dot product operation in MSA because we want every label in the dictionary to have an equal chance of being selected, and similar amplitude normalization operations are common in previous dictionary learning work. And we use the *Softmax* function to convert the similarity map S into the attention map A for subsequent calculations. Through this approach, our TDCGA is able to embed external priors into the learned dictionary to enhance input image features. We established the layer index (*l*) for both the input features and the token dictionary. Specifically, $X^{(l)}$ and $D^{(l)}$ represent the input features and the token dictionary of the *l*-th layer.

#### Spatial window self attention

**Fig. 4:**
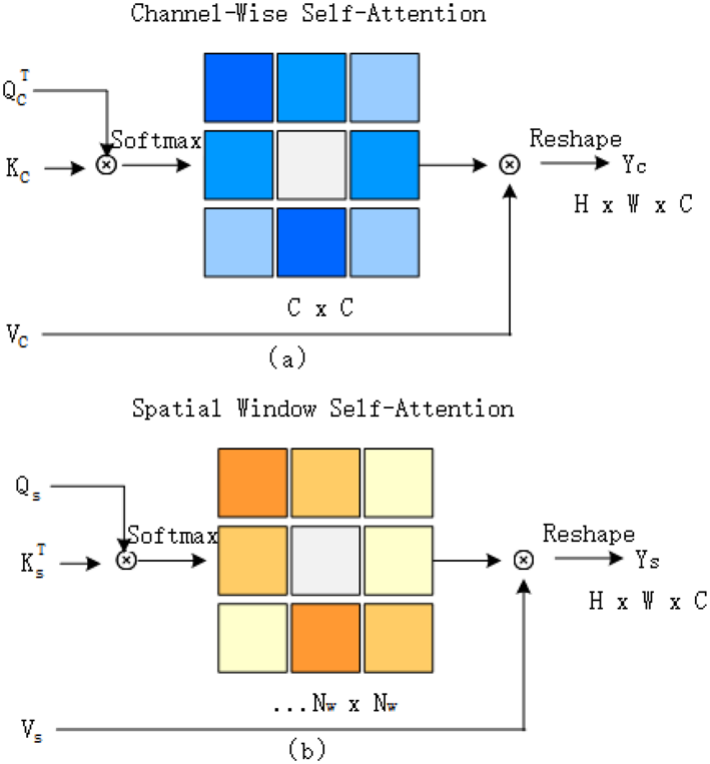
(**a**) Channel window self attention. (**b**) Spatial window self attention.

We put forward a mechanism called Spatial Window Self Attention (SW-SA), which focuses on calculating attention weights within specific windows. As illustrated in Fig. 4b, for the input feature X, its dimension is $R^{h \times w \times c}$,We first generate query (Q), key (K), and value (V) matrices through linear transformation. This step can be expressed as:

13 $$\begin{aligned} Q = XW_Q , \quad K = XW_K, \quad V = XW_V, \end{aligned}$$

where $W_Q, W_K, W_V \in {R}^{c \times c}$ are linear transformation matrixes that do not include bias terms. Next, we divide *Q*, *K*, and *V* into non-overlapping windows and flatten the features within each window into vectors. These flattened vectors are denoted as $Q_S, K_S, V_S$, with dimensions of ${R}^{H \times W \times C}$. Where $N_w$ represents the number of feature vector within each window. Then, we divide these vectors into h subsets, each corresponding to an attention head, represented as:

14 $$\begin{aligned} {\small {Q_S=[Q_1,\ldots ,Q_h] ,\quad K_S=[K_1,\ldots ,K_h] ,\quad V_S=[V_1,\ldots ,V_h]}}, \end{aligned}$$

The feature dimensions processed by each head are defined as $d = \frac{C}{h}$. The illustration in Fig. 4b is the situation with h = 1, where certain details are omitted for simplicity. The output $Y_s^i$ for the *i*-th head is defined as:

15 $$\begin{aligned} Y_s^i = \text {Softmax}\left(B + Q_s^i(K_s^i)^T/\sqrt{d}\right) \cdot V_s^i, \end{aligned}$$

where B represents the relative position encoding^44^. Finally, we obtain features $Y_s$ by reshaping and concatenating $Y_s^i$, with dimensions of $R^{h \times w \times c}$, expressed as:

16 $$\begin{aligned} Y_s & = concat\left(Y_s^1,\ldots , Y_s^h\right), \end{aligned}$$

17 $$\begin{aligned} {\text{SW-SA}}(X) & = Y_s W_p, \end{aligned}$$

where $W_P \in R^{c \times c}$ is a linear transformation matrix that integrates the features of all heads together. In addition, to capture richer spatial information, we adopted a shift window mechanism similar to that in Swin Transformer^37^.

#### Channel window self attention

We introduce a self-attention mechanism for the channel dimension, called channel window self attention (CW-SA). In this mechanism, we refer to previous research^45,46^ by dividing the channel into multiple independent heads and performing self-attention operations on each head separately. As shown in Fig. 4a, given input feature X, we first generate query (*Q*), key (*K*), and value (*V*) matrices through linear transformation, and reshape them to the size of $R^{hw \times c}$. These reshaped matrices are denoted as $Q_C$, $K_C$, and $V_C$. Similar to spatial window self attention (SW-SA), we divide these projection vectors into *h* parts, each corresponding to an attention head. To simplify the explanation, Fig. 4 shows the situation when $h = 1$. For the *i*-th head, the computation procedure for its channel self-attention can be articulated as:

18 $$\begin{aligned} Y_c^i = V_C^i \cdot Softmax\left(\left(Q_C^i)^T K_C^i / \beta \right)\right), \end{aligned}$$

where $Y_c^i \in R^{hw \times d}$ represents the output of the i-th head, *d* represents the dimension of the feature. $\beta$ is a learnable parameter used to adjust the scale of the inner product before the Softmax function. In the end, we concatenate all $Y_c^i$ and reshape them to obtain the final attention features $Y_c = R^{h \times w \times c}$. This process follows the same definition and equation as Eq. (16).

**Fig. 5:**
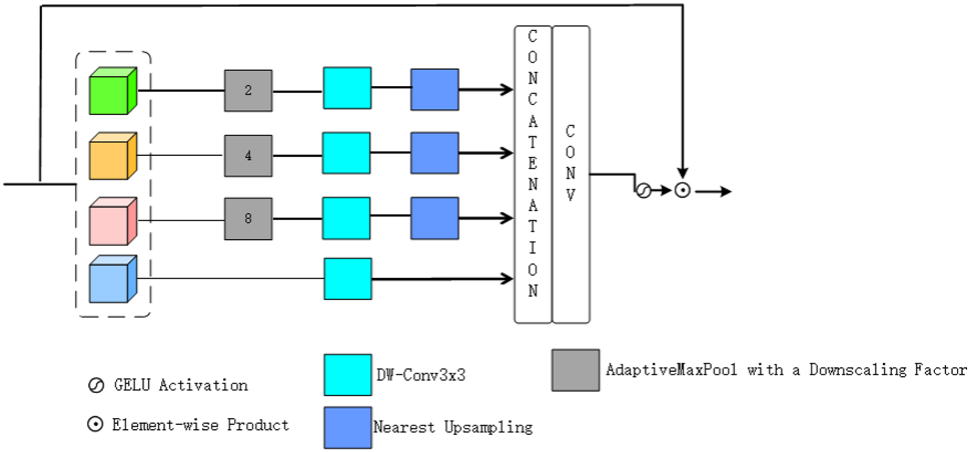
The proposed SSFM module structure diagram. Transform the LR image input by the convolutional layer into a feature space for feature extraction.

**Fig. 6:**
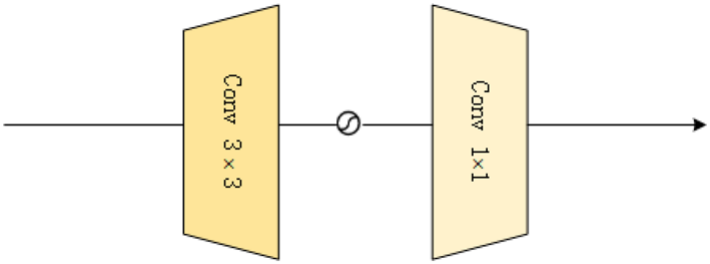
The proposed convolutional channel feature mixer consists of a $3\times 3$ convolution, a $1\times 1$ convolution, and an activation function.

**Table 1:** Quantitative comparison was conducted with the latest advanced methods.

Method	Modal (K)	Scale	Set5		Set14		B100		Urban100		Manga109	
			PSNR	SSIM	PSNR	SSIM	PSNR	SSIM	PSNR	SSIM	PSNR	SSIM
CARN^47^	1592	$\times$2	37.76	0.9590	33.52	0.9166	32.09	0.8978	31.92	0.9256	–	–
IMDN^48^	694	$\times$2	38.00	0.9605	33.63	0.9177	32.19	0.8996	32.17	0.9283	38.88	0.9774
SwinIR-light^25^	878	$\times$2	38.14	0.9611	33.86	0.9206	32.31	0.9012	32.76	0.9340	39.12	0.9783
LAPAR-A^49^	548	$\times$2	38.01	0.9065	33.62	0.9183	32.19	0.8999	32.10	0.9283	38.67	0.9772
ELAN-light^38^	582	$\times$2	38.17	0.9611	33.94	0.9207	32.30	0.9012	32.76	0.9340	39.12	0.9783
SwinIR-NG^50^	1181	$\times$2	38.17	0.9612	33.94	0.9205	32.31	0.9013	32.78	0.9340	39.20	0.9781
DAT-light^51^	553	$\times$2	38.24	0.9614	**34.01**	**0.9214**	32.34	0.9019	32.89	0.9346	39.49	0.9788
LKFN^52^	291	$\times$2	38.06	0.9609	34.00	0.9207	32.28	0.9011	32.92	0.9350	39.12	0.9779
MDRN^53^	304	$\times$2	38.11	0.9610	33.84	0.9205	32.32	0.9016	32.84	0.9350	39.14	0.9782
OSFFNet^54^	516	$\times$2	38.11	0.9610	33.72	0.9190	32.29	0.9012	32.67	0.9331	39.09	0.9780
HiT-SIR^55^	772	$\times$2	38.22	0.9613	33.91	0.9213	32.35	0.9019	**33.02**	**0.9365**	39.38	0.9782
DANS^56^	2456	$\times$2	*38.49*	*0.9622*	*34.28*	*0.9248*	*32.64*	*0.9039*	*33.58*	*0.9404*	*39.72*	*0.9796*
DBAN(ours)	849	$\times$2	**38.28**	**0.9616**	33.93	0.9207	**32.37**	**0.9020**	33.01	0.9355	**39.52**	**0.9789**
CARN^47^	1592	X3	34.29	0.9255	30.29	0.8407	29.06	0.8034	28.06	0.8493	33.50	0.9440
IMDN^48^	703	X3	34.36	0.9270	30.32	0.8417	29.09	0.8046	28.17	0.8519	33.61	0.9445
SwinIR-light^25^	918	X3	34.62	0.9289	30.54	0.8463	29.20	0.8082	28.66	0.8624	33.98	0.9478
LAPAR-A^49^	594K	X3	34.36	0.9267	30.34	0.8421	29.11	0.8054	28.15	0.8523	33.51	0.9441
ELAN-light^38^	590	X3	34.61	0.9288	30.55	0.8463	29.21	0.8081	28.69	0.8624	34.00	0.9478
SwinIR-NG^50^	1190	X3	34.64	0.9293	30.58	0.8471	29.24	0.8090	28.75	0.8639	34.22	0.9488
DAT-light^51^	561	X3	*34.76*	**0.9299**	30.63	**0.8474**	29.29	**0.8103**	28.89	0.8666	34.55	**0.9501**
LKFN^52^	299	X3	34.58	0.9286	30.51	0.8453	29.21	0.8081	28.70	0.8627	34.07	0.9476
MDRN^53^	311	X3	34.54	0.9284	30.54	0.8452	29.19	0.8079	28.74	0.8629	34.09	0.9476
OSFFNet^54^	524	X3	34.58	0.9287	30.48	0.8450	29.21	0.8080	28.49	0.8595	34.00	0.9472
HiT-SIR^55^	780	X3	34.72	0.9298	30.62	**0.8474**	29.27	0.8101	28.93	**0.8673**	34.40	0.9496
DANS^56^	2456	X3	33.96	*0.9329*	*30.88*	*0.8512*	*29.42*	*0.8132*	*29.31*	*0.8752*	*34.88*	*0.9519*
DBAN(ours)	851	X3	**34.73**	0.9298	**30.65**	**0.8474**	**29.30**	0.8102	**28.95**	**0.8673**	**34.57**	**0.9501**
CARN^47^	1592	$\times$4	32.13	0.8937	28.60	0.7806	27.58	0.7349	26.07	0.7837	30.47	0.9084
IMDN^48^	715	$\times$4	32.21	0.8948	28.58	0.7811	27.56	0.7353	26.04	0.7838	30.45	0.9075
SwinIR-light^25^	930	$\times$4	32.44	0.8976	28.77	0.7858	27.69	0.7406	26.47	0.7980	30.92	0.9151
LAPAR-A^49^	659	$\times$4	32.15	0.8944	28.61	0.7818	27.61	0.7366	26.14	0.7871	30.42	0.9074
ELAN-light^38^	601	$\times$4	32.43	0.8975	28.78	0.7858	27.69	0.7406	26.54	0.7982	30.92	0.9150
SwinIR-NG^50^	1201	$\times$4	32.44	0.8980	28.83	0.7870	27.73	0.7418	26.61	0.8010	31.09	0.9161
DAT-light^51^	573	$\times$4	**32.57**	0.8991	28.87	0.7879	27.74	0.7428	26.64	0.8033	31.38	0.9178
LKFN^52^	309	$\times$4	32.35	0.8971	28.80	0.7862	27.67	0.7400	26.60	0.8001	30.99	0.9140
MDRN^53^	322	$\times$4	32.35	0.8970	28.80	0.7861	27.69	0.7404	26.60	0.8005	31.02	0.9146
OSFFNet^54^	537	$\times$4	32.39	0.8976	28.75	0.7852	27.66	0.7393	26.36	0.7950	30.84	0.9125
HiT-SIR^55^	792	$\times$4	32.51	0.8991	28.84	0.7873	27.73	0.7424	26.71	0.8045	31.23	0.9176
DANS^56^	2456	$\times$4	*32.78*	*0.9028*	*28.98*	*0.7928*	*27.97*	*0.7468*	*27.32*	*0.8189*	*31.74*	*0.9228*
DBAN(ours)	854	$\times$4	**32.57**	**0.8993**	**28.92**	**0.7882**	**27.77**	**0.7429**	**26.85**	**0.8070**	**31.39**	**0.9184**

In this comparison, the top and second-best results are indicated in italic and bold, respectively.

### Feature aggregation module

#### Spatial split feature module (SSFM)

We propose a novel lightweight method aimed at capturing long-range dependencies from multi-scale features to optimize the reconstruction of HR images. As displayed in Fig. 5, we use a feature pyramid network^57^ to construct attention maps, which are used for spatially adaptive feature adjustment. with the purpose of reducing the complexity of the model and extract multi-scale features, we perform channel segmentation on the standardized input features to obtain four independent parts. Part of it is processed through $3\times 3$ deep convolution, while the rest is sent to the multi-scale feature generation module. Given the input feature X, the description of this process can be provided by the following formula:

19 $$\begin{aligned} & {[}X_0,X_1,X_2,X_3] = Split(X), \end{aligned}$$

20 $$\begin{aligned} & \hat{X_0} = DW-Conv_{3\times 3}(X_0), \end{aligned}$$

21 $$\begin{aligned} & \hat{X_i} = \uparrow _p\left(DW-Conv_{3\times 3}\left(\downarrow _\frac{p}{2^i}\left(X_i\right)\right)\right),\quad 1\le i\le 3, \end{aligned}$$

where $\uparrow _p$ represents upsampling features to the original resolution through nearest neighbor interpolation. $\downarrow _p$ Denotes downsampling features to the size of $\frac{p}{2^i}$. In order to filter out features that are helpful for learning non-local interactions, we apply adaptive max pooling on input features to generate multi-scale features. Next, we merge these multi-scale features and integrate local and global information through a $1\times 1$ convolution:

22 $$\begin{aligned} {\hat{X} = Conv_{1\times 1}\left(Concat\left([\hat{X_0}, \hat{X_1}, \hat{X_2}, \hat{X_3}]\right)\right),} \end{aligned}$$

After acquiring enhanced feature depictions $\hat{X}$,we standardize the data using the GELU non-linear activation method to determine the focus pattern and modify X in a responsive manner based on the focus pattern.

23 $$\begin{aligned} {\overline{X} = \phi (\hat{X}) \otimes X}, \end{aligned}$$

$\phi (\cdot )$represents the GELU function, and the operation $\otimes$ denotes element-wise multiplication. Leveraging multi-scale feature representation, we can apply this spatial adaptive modulation mechanism to collect remote features with less memory and computational cost. Compared with directly using deep convolution to extract features, this multi-scale form achieves better performance with less memory consumption.

**Table 2:** Examine the effect of the feature aggregation module on the final reconstruction outcomes for $\times 2$ SR on the Urban100 benchmark.

Baseline	FAM	Params (K)	PSNR (dB)	SSIM
$\checkmark$		751	32.86	0.9348
$\checkmark$	$\checkmark$	795	*32.88*	*0.9350*

Significant values are in italic.

**Table 3:** Examine the effect of the token dictionary cross-global attention module on the final reconstruction outcomes for $\times 2$ SR on the Urban100 benchmark.

Baseline	TDCGA	Params (K)	PSNR (dB)	SSIM
$\checkmark$		751	32.86	0.9348
$\checkmark$	$\checkmark$	800	*32.93*	*0.9352*

Significant values are in italic.

#### Convolutional channel feature mixer

We note that the first half of the FAM module focuses on exploring global information, while local contextual information is equally important for high-resolution image reconstruction. Unlike conventional feedforward networks, which typically use two consecutive $1\times 1$ convolutions to transform features and explore local contextual information, we are inspired by FMBConv^58^ and propose convolutional channel feature mixer (CCFM) to enhance local spatial modeling and perform channel mixing. As shown in Fig. 6, CCFM consists of a $3\times 3$ convolution and a $1\times 1$ convolution, where the $3\times 3$ convolution is used to encode spatial local context and double the number of channels for input features, followed by a $1\times 1$ convolution to reduce the number of channels back to the original dimension. The GELU function is applied to the hidden layer for nonlinear mapping, which is more memory efficient than using $3\times 3$ deep convolution (such as inverted residual blocks) on the extended dimension. The FAM module we propose can be represented as:

24 $$\begin{aligned} Y = LN( CCFM(SSFM(LN(X)))) , \end{aligned}$$

$LN(\cdot )$ represent LayerNorm layer, X represents the input feature, and Y denotes the output fused feature.

**Fig. 7:**
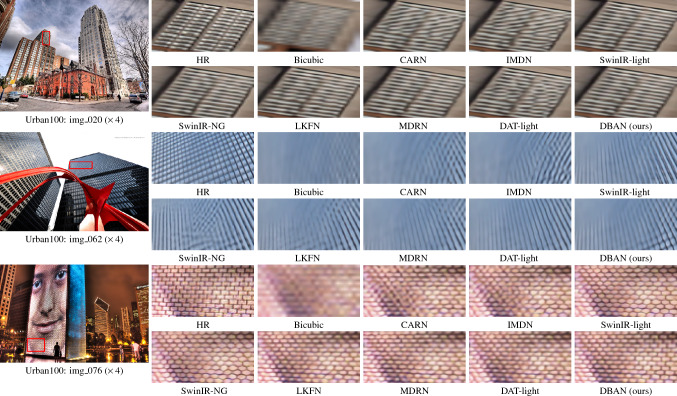
Visual results of compared methods on Urban100 dataset at a ($\times$4) SR setting.

**Table 4:** Model complexity comparisons ($\times 2$) for PSNR (dB) on Urban100 and Manga109, along with FLOPs and parameters, are reported.

Method	CARN^47^	SwinIR-light^25^	OSFFNet^54^	DiVANet^31^	SwinIR-NG^50^	HiT-SIR^55^	DBAN(ours)
Params(K)	1592	910	516	902	998	772	849
FLOPs(G)	222.8	243.7	83.2	189	140.4	209.9	167
Urban100	31.92	32.76	32.67	32.60	32.53	*33.02*	33.01
Manga109	38.36	39.12	39.09	39.08	38.97	39.38	*39.52*

Significant values are in italic.

## Experiment

**Fig. 8:**
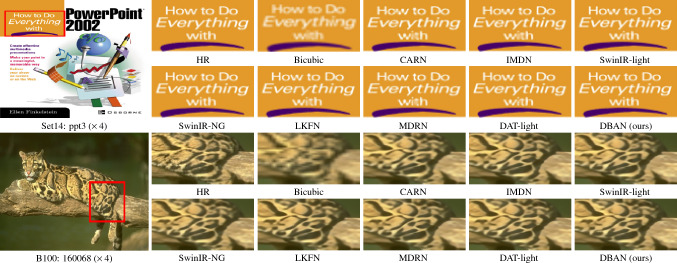
Visual results of cpmpared methods on Set14 and B100 dataset at a ($\times$4) SR setting.

**Fig. 9:**
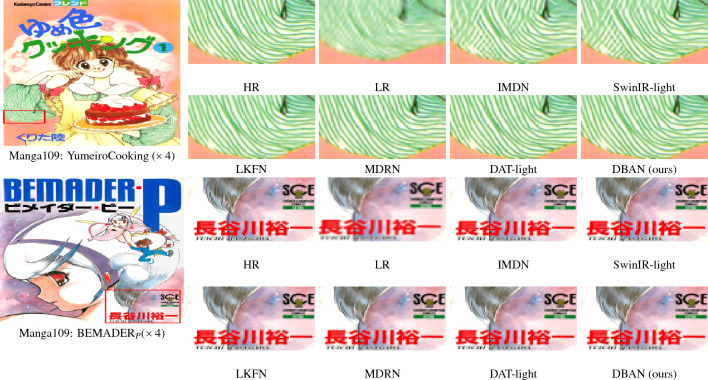
Comparative visualization for image SR at a $\times 4$ scale using the Manga109 dataset.

### Details of implementation and training

#### Datasets and evaluation metrics

In this research the DIV2K^59^ and Flickr2K^9^ datasets are used as the training sets, as well as five benchmark datasets: Set5^60^, Set14^61^, B100^62^, Urban100^63^, and Manga109^64^ for testing. Peak Signal to Noise Ratio (PSNR^65^) and Structural Similarity (SSIM^66^) are used as evaluation metrics in these datasets for model performance assessment. To ensure a fair comparison with other SR techniques, we transform the image from RGB to YCbCr format and subsequently compute the PSNR and SSIM on the Y channel.

#### Training setting

For DBAN, there are 4 residual groups(RGs), and each RG contains 6 TAB modules. At the same time, there are also 2 FAM modules. We adhere to the majority of prior research in training and testing our model. We train the model using patches of size $64\times 64$ and a batch size set to 16. Horizontal flipping and random rotations of $90^\circ$, $180^\circ$, and $270^\circ$ are used for data augmentation while model training. We created 64 tokens for the external dictionary *D* in each TAB block and set the reduction rate $r=6$. We use the AdamW^67^ optimizer with $\beta _1=0.9$ and $\beta _2=0.9$ to minimize L1 pixel loss estimation between HR and ground truth. The learning rate was initially set to $2\times 10^{-4}$, and then decreased by half after the 250th, 400th, 450th, and 475th epochs. All training and testing of the model were conducted using PyTorch^68^ on the RTX 4090 GPU.

### Comparison with state-of-the-art methods

We have compared our model with the current 11 state-of-the-art image super-resolution methods: CARN^47^, IMDN^48^, SwinIR-light^25^, LAPAR-A^49^, ELAN-light^38^, SwinIR-NG^50^, DAT-light^51^, LKFN^52^, MDRN^53^. OSFFNet^54^ HiT^55^. Table 1 shows quantitative comparison, while Figs. 7, 8 and 9 provides visual comparison.

#### Quantitative results

Table 1 shows the results of image SR on $\times$2, $\times$3, and $\times$4 factors. on the Manga109 dataset at a $\times$4 scale, our method achieves a PSNR value that is 0.97 dB higher than that of LAPAR-A^49^. Similarly, in the Urban100 dataset at a $\times$3 scale, our method’s PSNR value exceeds that of CARN^47^ by 0.89dB. Our DBAN outperforms almost all comparison methods on the benchmark dataset in three aspects.

#### Visual results

As shown in Fig. 7, we provide the visualization comparison results magnified by a factor of $\times 4$ on the Urban100 dataset. We have presented the visualization comparison results at a $\times 4$ magnification on the Set14, B100, and Manga109 datasets in Figs. 8 and 9, respectively. In contrast, our method distinguishes itself by effectively mitigating these artifacts, which are often attributed to the limitations of conventional upgrading techniques. For instance, in img_020, most comparative methods struggle to restore details and introduce unwanted artifacts. Nevertheless, our DBAN is capable of restoring the correct structure with distinct textures. Similar observations can be made in img_062 and img_076. This is primarily due to our method’s enhanced representational capabilities, stemming from the extraction of complex features from various dimensions.

### Ablation study

#### Feature aggregation module

To illustrate the impact of the feature aggregation module, we conducted an ablation study in Table  2, comparing the results with and without the feature aggregation module at Urban100 scaling factor $\times$2. Not using feature aggregation module Compared with the results, using the feature aggregation module increased PSNR by 0.02.

#### Token dictionary cross global attention

We confirmed its effectiveness by carrying out ablation studies on the cross global attention of the token dictionary. In Table 3, we chose a scaling factor ($\times 2$) to assess the performance of token dictionary cross global attention. The results on Urban100 with a scaling factor of ($\times 2$) showed that compared to the baseline model, PSNR was increased by 0.07 dB through the addition of token dictionary cross global attention. The ablation experiment shows that the proposed token dictionary cross global attention can improve the the model’s global modeling capability and yield high-quality HR images (Table 4).

#### The impact of the number of residual groups and attention heads on model performance

We explored the impact of different RGs and the number of attention heads on the final network reconstruction performance through ablation experiments. As shown in Table 5, we set the number of RGs and attention heads to 2, 4, and 6 for training. The final test results of the five benchmark scaling factors of $\times 2$ indicate that the final reconstruction improves with the increase of residual group and attention head. This confirms that the attention module in the RGs can improve the final network performance, thereby enhancing its ability to model global images. It is evident from the table that the performance of the model improves with the increase of RGs and attention heads. However, in order to balance computational cost and reconstruction performance, we chose RGs = 4 and attention heads = 4 in the final network.

**Table 5:** The investigation of the effectiveness of different residual groups and attention heads on reconstruction performance.

Parameter	Set5	Set14	BSD100	Urban100	Manga109
$RGs = 2$	37.53	33.21	31.93	32.17	38.58
$RGs = 4$	38.28	**33.93**	32.37	**33.01**	**39.52**
$RGs = 6$	**38.29**	**33.93**	**32.38**	32.99	**39.52**
$heads=2$	37.86	33.54	32.05	32.48	39.05
$heads=4$	**38.28**	**33.93**	32.37	33.01	39.52
$heads=6$	38.26	33.90	**32.41**	**33.03**	**39.56**

Average PSNR (dB) when the scaling factor is $\times$2 on different benchmarks. The best performance is shown in bold.

#### Performance variations with different token dictionary sizes (M)

In our experiments, we investigate the impact of token dictionary size $M$ on model performance by varying $M$ from 16 to 96. As shown in Table 6, the results demonstrate that increasing $M$ initially improves performance, with PSNR and SSIM scores peaking at $M = 64$ (26.55 and 0.7977 on Urban100; 30.99 and 0.9144 on Manga109). However, further increasing $M$ to 96 leads to a slight performance degradation, indicating that an excessively large dictionary may exceed the model’s capacity and introduce redundancy. These findings suggest that an optimal token dictionary size exists, balancing expressiveness and computational efficiency.

**Table 6:** Ablation study on token dictionary sizes (M).

M	Urban100		Manga109	
	PSNR	SSIM	PSNR	SSIM
16	26.41	0.7949	30.91	0.9137
32	26.47	0.7963	30.95	0.9140
64	**26.55**	**0.7977**	**30.99**	**0.9144**
96	26.54	0.7975	30.95	0.9142

Significant values are in bold.

### Model size analysis

To evaluate the model’s efficiency, we compare the number of parameters with several state-of-the-art algorithms. Additionally, we tested the floating-point operations per second (FLOPS) and parameter counts of our model on the same RTX 4090 GPU with a scaling factor of $\times 2$. In Table 4, our model has roughly half the number of parameters and approximately 55G fewer floating-point operations compared to CARN^47^, while achieving a 0.16dB higher PSNR on the Manga109 dataset.

### Analysis of training time and memory usage

On the RTX 4090 GPU, we simulated 50 rounds of training and 1000 instances of inference with a scaling factor of 4. The optimizer used Adam, and the learning rate was set at $1\times 10^{-3}$. As shown in Table 7, during the training phase, the average time per epoch was 268.21 ms, with a maximum memory usage of 1936.48 MB. In the inference phase, the average inference time was 45.9 ms, and the maximum memory usage was 56.34 MB. This indicates that our model is efficient in terms of training and inference time, while consuming minimal memory.

**Table 7:** Analysis on the time and memory consumption of training and inferring $\times 4$ model on RTX4090.

Stage	#Avg. inference time	#Avg. training time	# Max. memory usage (MB)
Training	–	268.21 ms	1936.48
Inference	45.9 ms	–	56.34

**Table 8:** Comparison of different methods in terms of GPU memory usage and average processing time.

Methods	#GPU Mem. (M)	#Avg. time (ms)
CARN-M^47^	680.84	17.85
CARN^47^	689.83	18.90
EDSR-baseline^9^	486.58	19.81
IMDN^48^	203.44	10.22
LAPAR-A^49^	1811.47	24.91
Ours	4600.3	63.02

### Memory and running time comparisons

To comprehensively evaluate the performance of our suggested approach, we conducted a comparison with five representative methods: CARN-M^47^, CARN^47^, EDSR-baseline^9^, IMDN^48^, and LAPAR-A^49^. We evaluated GPU memory consumption (#GPU Mem.) and runtime (#Avg. Time) at a $\times$4 super-resolution (SR) scale. During inference, we recorded the peak GPU memory consumption. The runtime was calculated as the average over 50 test images, each with a resolution of 320 $\times$ 180 pixels. Table 8 presents a comparison of our method’s memory usage and runtime with those of the other methods. In terms of memory usage, our DBAN is approximately 20 times larger than IMDN^48^ and about 9 times larger than EDSR^9^. However, its running speed is only 6 times that of IMDN^48^ and 3 times that of EDSR^9^. Tables 4 and 8 illustrate that the model we have introduced provides an advantageous balance among the speed of inference, the intricacy of the model, and the quality of reconstruction outcomes, compared to state-of-the-art methods.

## Conclusion

In this work, we introduce a novel dual-stream multitasking model for efficient image super-resolution. Our model, DBAN, aggregates channel and spatial features through a dual approach of intra-block and inter-block processing, demonstrating robust representation capabilities. Drawing inspiration from traditional dictionary learning methods, we propose a token dictionary cross global attention mechanism that provides external additional information, which helps to enrich any lacking high-quality detail features. Consequently, we not only harness local features but also merge global features, overcoming the constraints of local windows and establishing remote connections between similar structures within the image. Furthermore, we propose modeling global dependencies using spatial window attention and channel window attention, achieving intra-block feature aggregation across both spatial and channel dimensions. In the image reconstruction section, we introduce Dysample to replace the traditional pixelshuffle, aiming for a more lightweight architecture. Extensive experiments have demonstrated that DBAN delivers superior performance with reduced complexity.

## Data Availability

The datasets generated and/or analysed during the current study are available in the https://github.com/igotsmoke9/DBAN.
